# A scoping review of Enhanced Recovery After Surgery (ERAS), protocol implementation, and its impact on surgical outcomes and healthcare systems in Africa

**DOI:** 10.1186/s13741-024-00435-2

**Published:** 2024-08-02

**Authors:** Fitsum Kifle, Peniel Kenna, Selam Daniel, Salome Maswime, Bruce Biccard

**Affiliations:** 1 Global Surgery Division, Department of Surgery, Faculty of Health Sciences, University of Cape Town, Cape Town, South Africa; 2 Network for Perioperative and Critical Care, Debre Birhan University Asrat Woldeyes Health Sciences Campus, Debre Birhan, Ethiopia; 3 Department of Anesthesiology and Critical Care, Kidus Petros Hospital, Addis Ababa, Ethiopia; 4 Department of Anaesthesia and Perioperative Medicine, Groote Schuur Hospital, University of Cape Town, Cape Town, Western Cape South Africa

**Keywords:** ERAS, Africa, Outcomes, Review

## Abstract

**Background:**

Enhanced Recovery After Surgery (ERAS) is a patient-centered approach to surgery designed to reduce stress responses and facilitate faster recovery. ERAS protocols have been widely adopted in high-income countries, supported by robust research demonstrating improved patient outcomes. However, in Africa, there is limited evidence regarding its implementation. This review aims to identify the existing literature on the implementation of ERAS principles in Africa, the reported clinical outcomes, and the challenges and recommendations for successful implementation.

**Methods:**

We conducted a librarian-assisted literature search of electronic research databases between October and November 2023. Titles and abstracts were screened for eligibility, and duplicates were then removed, followed by full-text assessment of potentially eligible studies. We utilized the summative content analysis method to synthesize and group the data into fewer categories based on agreed-upon criteria. Descriptive statistics were used to describe the results.

**Results:**

The search identified 342 potential studies resulting in 15 eligible studies for inclusion in the review. The publication years ranged from 2016 to 2023. The studies originated from three countries: Egypt (*n* = 10), South Africa (*n* = 4), and Uganda (*n* = 1). Successful implementation was associated with reduced hospital length of stay (*n* = 12), lower mortality rates (*n* = 3), and improved pain outcomes (*n* = 7). Challenges included protocol adherence (*n* = 5) and limitations of the research design to generate strong evidence (*n* = 3). Recommendations included formal adoption of ERAS principles (*n* = 5), the need for sustained research commitment, and exploration of the applicability of ERAS in diverse surgical contexts (*n* = 8). Large-scale implementation beyond individual institutions was encouraged to further validate its impact on patient outcomes and healthcare costs (*n* = 1).

**Conclusions:**

Despite the limited number of studies on ERAS implementation in Africa, the available evidence suggests that it reduces the length of hospital stays and mortality rates. This is crucial for the region, given its higher mortality rates, necessitating more collaborative, methodically well-designed studies to establish stronger evidence for ERAS in lower-resource environments.

**Supplementary Information:**

The online version contains supplementary material available at 10.1186/s13741-024-00435-2.

## Background

The Enhanced Recovery After Surgery (ERAS) concept was introduced in the early 1990s based on improved understanding of the pathophysiology of postoperative recovery (Kehlet 2023; Subramaniam and Horgan 2023; Ljungqvist et al. 2024). ERAS is a comprehensive approach to perioperative care that aims to reduce the stress response to surgery, enabling a faster recovery and return to baseline function (Ljungqvist et al. 2024; Teeter et al. 2023; Taurchini et al. 2018). Implementing ERAS protocols has shown significant benefits in various surgical specialties, leading to improved patient satisfaction and reduced healthcare costs (Senturk et al. 2023). Over the past three decades, ERAS has gained popularity and has been widely adopted in hospitals worldwide (Kehlet 2023; Ljungqvist et al. 2024). Its holistic approach to patient management is not only improving patient outcomes but also fostering collaboration between healthcare disciplines, resulting in a more efficient and patient-centered surgical experience.

In high-income countries, ERAS has become the standard of care for various surgical procedures, supported by specialty-specific guidelines and protocols (Ljungqvist et al. 2024; Taurchini et al. 2018; McQueen et al. 2023; Turaga 2023). The adoption of ERAS practices is supported by a strong ERAS societal establishment and evidence-based research (Taurchini et al. 2018; Home - ERAS® Society 2023). The ERAS society provides continuous education and support to health care professionals, ensuring that they remain current with ERAS protocols (Home - ERAS® Society 2023; Ljungqvist 2023). The society helps validate the effectiveness of ERAS practices, further encouraging their widespread adoption in surgical settings. Currently, the society is active in over 20 countries, and evidence from these member states demonstrates that ERAS protocols have significantly improved patient outcomes, including reduced complications and shorter hospital stays (Ljungqvist et al. 2024; Home - ERAS® Society 2023). This evidence supports the global expansion of ERAS implementation.

However, evidence on the implementation of ERAS practice in Africa is very limited (Su et al. 2000), and most reports from the region are small-scale studies or anecdotal reports. Additionally, resource constraints may further hinder the widespread implementation of ERAS in this region. In this scoping review, we aimed to identify and analyze the existing literature on the implementation of ERAS practice in Africa. By examining the current evidence, we hope to gain insights into the challenges and opportunities for implementing ERAS in this region, as well as identify potential strategies for overcoming barriers and promoting successful adoption of ERAS protocols.

## Method

This scoping review followed Joanna Briggs Institute (JBI’s) guidelines for scoping reviews (Peters et al. 2023a). The Preferred Reporting Items for Systematic Reviews and Meta-Analyses extension for Scoping Reviews (PRISMA-ScR) was used for reporting (Tricco et al. 2018). This review was conducted in accordance with an a priori protocol registered in the Open Science Framework 10.17605/OSF.IO/D4N52.

### Review questions

1. What is the extent of literature available on the implementation of ERAS protocols in African healthcare settings?
2. What are the common reported practices and clinical outcomes associated with the adoption of ERAS protocols in various surgical specialties in Africa?
3. What are the challenges in the implementation of ERAS protocols within the African context?
4. What are the recommendations for the successful implementation of ERAS protocols in African surgical practice?
5. Are there gaps in the current research, and what areas require further investigation to understand the impact of ERAS protocols on surgical outcomes in African populations?

### Search strategy

An independent librarian-assisted systematic search of electronic databases, including PubMed, Scopus, Embase, Cochrane, and Web of Sciences, was conducted between October and November 2023. The search strategy included keywords related to Africa, names of African countries, ERAS, and fast-track surgery, as shown in Appendix 1.

#### Inclusion and exclusion criteria

##### Inclusion criteria

The inclusion criteria for the study were defined using the updated methodological guidance for the conduct of scoping reviews using the “population, context, and concept” (PCCs) framework (Peters et al. 2023b). The population included patients and healthcare providers in African healthcare settings. The context included healthcare settings providing surgery across Africa. The central concept investigated was the implementation and impact of ERAS protocols across various surgical specialties within African healthcare settings.

The inclusion criteria were therefore defined as follows:

1. Publication type: Peer-reviewed journal articles
2. Geographic focus: Studies conducted in any African country or healthcare setting
3. Time period: Time filter was not applied to include all relevant studies.
4. Language: Studies published in English or, if available, with English translations or abstract
5. Topic relevance: Studies exploring the implementation and impact of ERAS protocols in surgical practice within the African context.

The exclusion criteria were as follows:

1. Publication type: Conference abstracts, letters, editorials, and commentaries
2. Geographic focus: Studies conducted outside of Africa
3. Time period: Date and time filters were not applied.
4. Language: Studies published in languages other than English, with no available translations
5. Topic irrelevance: Studies that do not focus on the implementation and impact of ERAS protocols on surgical practice within the African context

### Data screening and analysis

All studies were imported into the Mendeley software, and duplicates were removed. All studies were assessed in duplicate for eligibility by FK, PK, and SD, who also performed full-text assessments of potentially eligible studies following the screening of titles and abstracts for eligibility. The agreement on the full-text assessments was based on the pre-established inclusion and exclusion criteria of the study, listed above. In cases of disagreement, the matter was referred to S. M. and B. B. for comprehensive evaluation to ensure the inclusion of relevant research. We extracted data on ERAS implementation and the reported outcomes, challenges encountered, and recommendations on ERAS implementation data for our study, as shown in Appendix 2.

Data analysis was conducted using Google spreadsheet to organize and summarize the relevant studies based on the specified criteria. The variables from included publications were extracted to the spreadsheet, which included titles, authors, publication year, journal name, country of study, study design, language of study, and topic of relevance. Data synthesis was performed on the topic of relevance, geographic focus, reported clinical outcomes, challenges in adopting, and recommendations associated with the implementation of ERAS practice in African settings. We utilized the summative content analysis method to synthesize and group these data into fewer categories based on agreed-upon and predefined criteria (Hsieh and Shannon 2005). Descriptive statistics were used to present the characteristics of the included studies, and tables were used to present the findings.

### Definitions

The following are the predefined criteria for our review:

- *Clinical outcomes after implementation*: Refer to the observable effects and results of implementing ERAS protocols, as reported by the investigators.
- *Challenges encountered in implementing the ERAS protocol*: Refer to obstacles or difficulties faced during the adoption and execution of ERAS guidelines, as described by the investigators.
- *Recommendations proposed*: Refer to suggested actions or strategies proposed to address the challenges identified in implementing ERAS protocols.

## Results

**Fig. 1:**
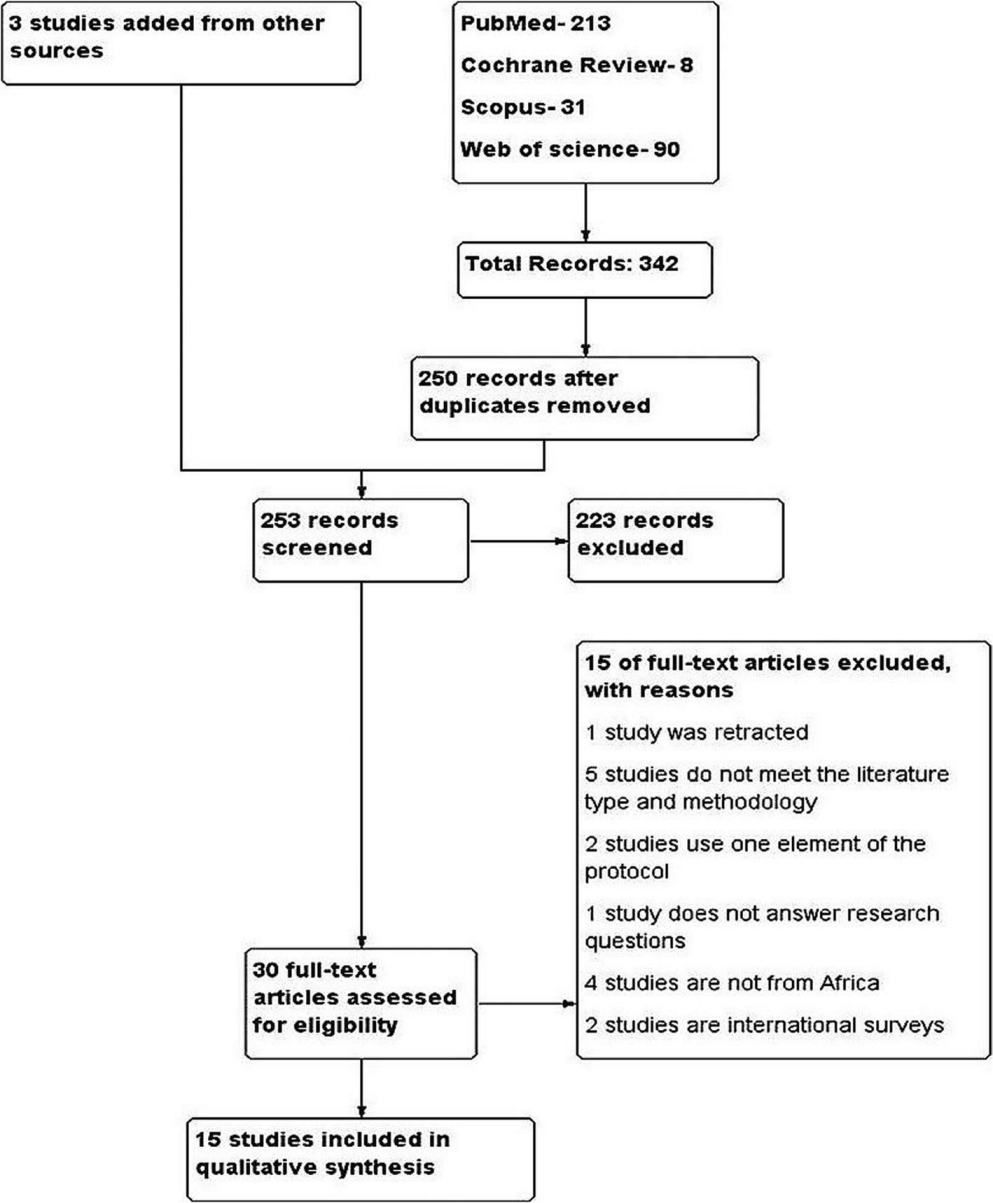
PRISMA workflow diagram

The database search identified 342 potential studies. After removal of duplicates, title and abstract review, citation search, and full-text review, 12 eligible studies were identified. A search of the reference lists of the eligible studies identified an additional 3 studies, with a total of 15 studies included in the analysis. The search results and inclusion and exclusion processes are shown in Fig. 1.

### Characteristics of included studies

Fifteen articles were included which evaluated the implementation of ERAS protocols across various surgical specialties in African healthcare settings. The surgical procedures included gastrointestinal (Fathy et al. 2023), bariatric (Loots et al. 2023), colorectal (Oodit et al. 2021), radical cystectomy (Ammar et al. 2023), total hip and knee arthroplasty (Plenge et al. 2023; Beukes et al. 2022), emergency cesarean deliveries (Baluku et al. 2023), kidney transplantation (Elsabbagh et al. 2023), abdominal hysterectomy (*n* = 3) (Ferghali et al. 2023; Mohamed Ibrahim et al. 2023; Ahmed et al. 2023), spinal surgery (Elgamal et al. 2023), and gynecologic (Abdelrazik and Sanad 2023) and gynecologic oncology (*n* = 2) (Sarhan et al. 2023; Sameer et al. 2023).

**Fig. 2:**
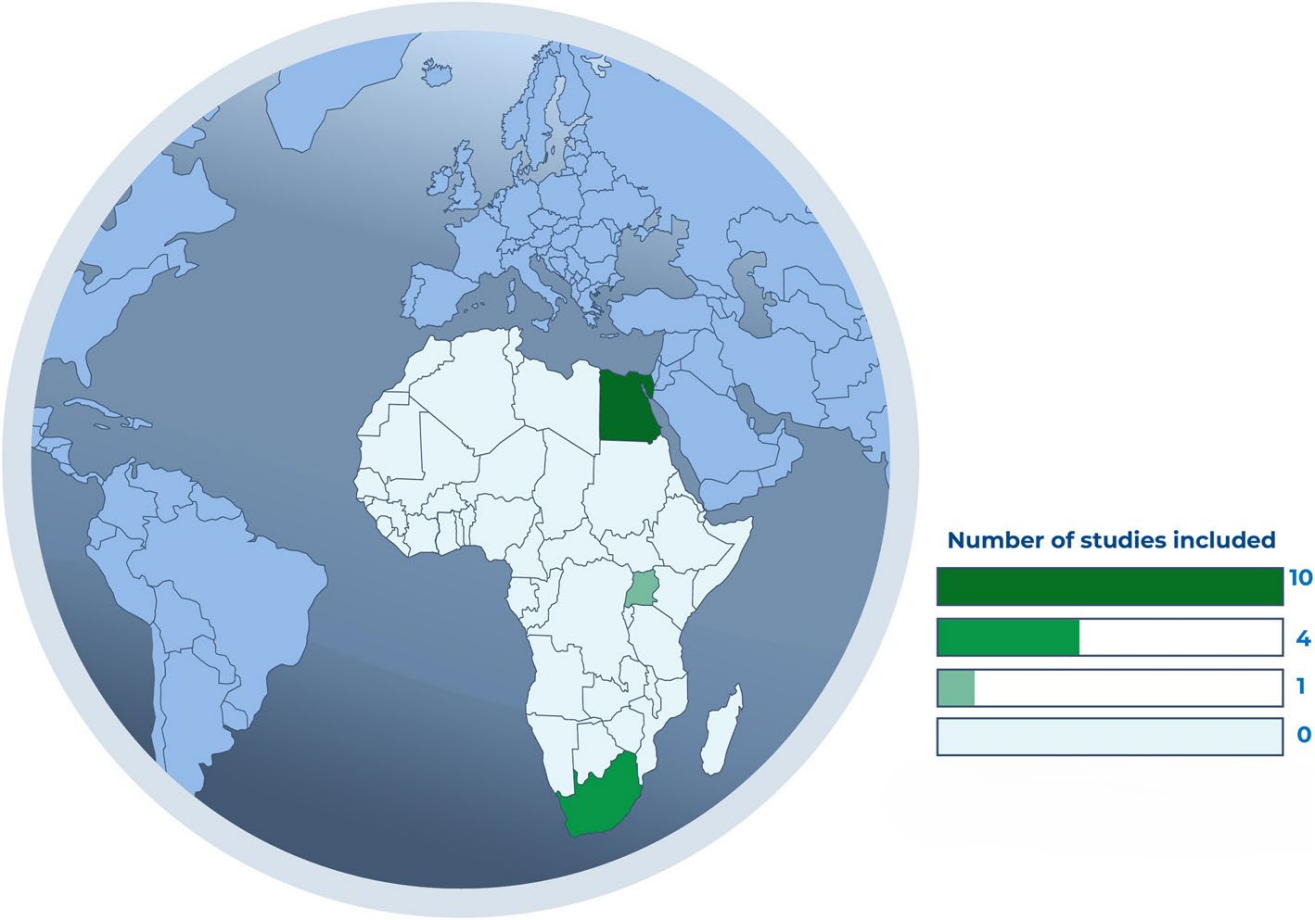
ERAS implementation studies in Africa: geographical distribution overview

The publications were from three African countries: Egypt (*n* = 10) (Fathy et al. 2023; Ammar et al. 2023; Elsabbagh et al. 2023; Ferghali et al. 2023; Mohamed Ibrahim et al. 2023; Ahmed et al. 2023; Elgamal et al. 2023; Abdelrazik and Sanad 2023; Sarhan et al. 2023; Sameer et al. 2023), South Africa (*n* = 4) (Loots et al. 2023; Oodit et al. 2021; Plenge et al. 2023; Beukes et al. 2022), and Uganda (*n* = 1) (Baluku et al. 2023) published between 2016 and 2023, as the geographical distribution is shown in Fig. 2. The research methodologies included prospective cohorts (*n* = 6) (Fathy et al. 2023; Loots et al. 2023; Oodit et al. 2021; Ammar et al. 2023; Plenge et al. 2023; Sarhan et al. 2023), randomized controlled trials (RCT) (*n* = 4) (Baluku et al. 2023; Ferghali et al. 2023; Elgamal et al. 2023; Abdelrazik and Sanad 2023), retrospective cohorts (*n* = 2) (Beukes et al. 2022; Elsabbagh et al. 2023), non-randomized clinical trials (*n* = 1) (Sameer et al. 2023), and quasi-experimental designs (*n* = 2) (Mohamed Ibrahim et al. 2023; Ahmed et al. 2023). A further description of the characteristics of the included studies is shown in Table 1.

**Table 1:** Characteristics of the included studies

Title	Year of publication	Journal	Country	Surgical specialty	Sample size	Type of research
Enhanced recovery protocols versus traditional methods after resection and reanastomosis in gastrointestinal surgery in pediatric patients (Hsieh and Shannon 2005)	2018	*Annals of Pediatric Surgery*	Egypt	Gastrointestinal surgery	60	Prospective cohort
The successful implementation of a modified Enhanced Recovery After Surgery (ERAS) program for bariatric surgery in a South African teaching hospital (Fathy et al. 2023)	2018	*Surgical Laparoscopy Endoscopy & Percutaneous Techniques*	South Africa	Bariatric surgery	62	Prospective cohort
Implementation of Enhanced Recovery After Surgery for endometrial carcinoma: a non-randomized controlled trial (Sarhan et al. 2023)	2019	*BMJ*	Egypt	Endometrial carcinoma	58	Non-randomized clinical trial
Quality of recovery after total hip and knee arthroplasty in South Africa: a national prospective observational cohort study (Ammar et al. 2023)	2020	*BMC Musculoskeletal Disorders*	South Africa	Total hip and knee arthroplasty	186	Prospective cohort
Implementation of Enhanced Recovery After Surgery in gynecological operations: a randomized controlled trial (Elgamal et al. 2023)	2020	*Ain-Shams Journal of Anesthesiology*	Egypt	Gynecological operations	216	RCT
Implementation of Enhanced Recovery After Surgery as a protocol versus routine care on women undergoing hysterectomy (Elsabbagh et al. 2023)	2020	*Assiut Scientific Nursing Journal*	Egypt	Abdominal hysterectomy	140	RCT
Improvement of outcome by implementation of enhanced recovery pathway in gynecologic/oncologic surgery (Abdelrazik and Sanad 2023)	2021	*EJHM*-	Egypt	Gynecological oncologic surgeries	54	Prospective cohort
Colorectal surgical outcomes following implementation of an Enhanced Recovery After Surgery program in Cape Town (Loots et al. 2023)	2021	*South African Journal of Surgery*	South Africa	Colorectal	457	Prospective cohort
The efficacy of enhanced recovery protocol from anesthesia in diabetic patients undergoing radical cystectomy (Oodit et al. 2021)	2021	*Alexandria Journal of Medicine*	Egypt	Radical cystectomy	54	Prospective cohort
A randomized controlled trial of Enhanced Recovery After Surgery versus standard of care recovery for emergency cesarean deliveries at Mbarara Hospital, Uganda (Beukes et al. 2022)	2022	*Anesthesia & Analgesia*	Uganda	Emergency cesarean deliveries	160	RCT
Comparing outcomes between Enhanced Recovery After Surgery and traditional protocols in total knee arthroplasty: a retrospective cohort study (Plenge et al. 2023)	2022	*South African Orthopaedic Journal*	South Africa	Total knee arthroplasty	119	Retrospective cohort
Enhanced Recovery After Surgery pathway in kidney transplantation: the road less traveled (Baluku et al. 2023)	2022	*Transplantation Direct*	Egypt	Kidney transplantation	20	Retrospective cohort
Effect of Enhanced Recovery After Surgery protocol on postoperative outcomes of women undergoing abdominal hysterectomy (Ferghali et al. 2023)	2023	*SAGE Open Nursing*	Egypt	Abdominal hysterectomy	118	Quasi-experimental design
Enhanced recovery after spinal surgery protocol versus conventional care in non-insulin diabetic patients: a prospective randomized trial (Ahmed et al. 2023)	2023		Egypt	Spinal surgery	72	RCT
Effect of enhanced recovery nursing program on recovery process of women after hysterectomy operation in Suez Canal University Hospital and General Hospital at Ismailia City (Mohamed Ibrahim et al. 2023)	2023	*IJNRHN*	Egypt	Abdominal hysterectomy	132	Quasi-experimental study

Keys: *BMC* Biomed Central, *BMJ* British Medical Journal, *EJHM* Egyptian Journal of Hospital Medicine, *IJNRHN* International Journal of Novel Research in Healthcare and Nursing, *RCT* randomized control trial, *SAMJ* South African Medical Journal

### Findings of the review

#### A. Reported clinical outcomes

The reported clinical outcomes of ERAS implementation include a reduction in hospital length of stay (*n* = 12) (Fathy et al. 2023; Loots et al. 2023; Ammar et al. 2023; Plenge et al. 2023; Beukes et al. 2022; Baluku et al. 2023; Ferghali et al. 2023; Mohamed Ibrahim et al. 2023; Abdelrazik and Sanad 2023; Sarhan et al. 2023; Sameer et al. 2023; Oodit et al. 2018), lower mortality rates (*n* = 3) (Loots et al. 2023; Ammar et al. 2023; Ahmed et al. 2023), lower pain scores and improved pain management (*n* = 7) (Plenge et al. 2023; Beukes et al. 2022; Baluku et al. 2023; Ahmed et al. 2023; Elgamal et al. 2023; Abdelrazik and Sanad 2023; Sarhan et al. 2023), and a decrease in hospital readmissions (*n* = 2) (Ammar et al. 2023; Abdelrazik and Sanad 2023). Implementation was also associated with a decrease in morbidity (*n* = 6) (Loots et al. 2023; Plenge et al. 2023; Ahmed et al. 2023; Abdelrazik and Sanad 2023; Sameer et al. 2023; Oodit et al. 2018) and an increase in early mobility (*n* = 4) (Baluku et al. 2023; Ahmed et al. 2023; Elgamal et al. 2023; Abdelrazik and Sanad 2023).

Improvement in the functional scores (*n* = 1) (Ahmed et al. 2023), patient satisfaction (*n* = 1) (Ahmed et al. 2023), early rehabilitation (*n* = 1) (Ahmed et al. 2023), and the prompt initiation of oral feeding (*n* = 3) (Oodit et al. 2021; Elgamal et al. 2023; Abdelrazik and Sanad 2023) were also reported following the implementation of ERAS. ERAS was associated with cost savings (*n* = 4) (Plenge et al. 2023; Ahmed et al. 2023; Sarhan et al. 2023; Sameer et al. 2023), optimizing antibiotic use (*n* = 1) (Oodit et al. 2021), refining fluid management (*n* = 2) (Beukes et al. 2022; Ferghali et al. 2023), and improved overall quality of recovery (*n* = 2) (Beukes et al. 2022; Sarhan et al. 2023). Table 2 further describes the reported clinical outcomes of ERAS implementation.

**Table 2:** Reported clinical outcomes

Clinical outcome of ERAS protocol	Number of articles
Reduced hospital length of stay (Peters et al. 2023b; Hsieh and Shannon 2005; Fathy et al. 2023; Loots et al. 2023; Oodit et al. 2021; Ammar et al. 2023; Plenge et al. 2023; Baluku et al. 2023; Elsabbagh et al. 2023; Ahmed et al. 2023; Elgamal et al. 2023; Abdelrazik and Sanad 2023)	12
Reduced pain score and improved management (Oodit et al. 2021; Ammar et al. 2023; Plenge et al. 2023; Ferghali et al. 2023; Mohamed Ibrahim et al. 2023; Ahmed et al. 2023; Elgamal et al. 2023)	7
Reduced morbidity (Hsieh and Shannon 2005; Oodit et al. 2021; Elsabbagh et al. 2023; Ahmed et al. 2023; Abdelrazik and Sanad 2023; Sarhan et al. 2023)	6
Early mobility (Plenge et al. 2023; Ferghali et al. 2023; Mohamed Ibrahim et al. 2023; Ahmed et al. 2023)	4
Lowered overall costs (Oodit et al. 2021; Ferghali et al. 2023; Elgamal et al. 2023; Abdelrazik and Sanad 2023)	4
Reduced mortality (Hsieh and Shannon 2005; Loots et al. 2023; Ferghali et al. 2023)	3
Early initiation of oral feeding (Fathy et al. 2023; Mohamed Ibrahim et al. 2023; Ahmed et al. 2023)	3
Reduced readmission (Loots et al. 2023; Ahmed et al. 2023)	2
Decrease in IV fluid requirement (Ammar et al. 2023; Baluku et al. 2023)	2
Improved quality of recovery (Ammar et al. 2023; Elgamal et al. 2023)	2
Improvement in functional scores (Ferghali et al. 2023)	1
Reduced catheterization duration (Plenge et al. 2023)	1
Improved patient satisfaction (Ferghali et al. 2023)	1
Early rehabilitation (Ferghali et al. 2023)	1
Better postoperative follow-up (Hsieh and Shannon 2005)	1
Reduced postoperative fever and chest infection (Peters et al. 2023b)	1
Shorter duration of antibiotic use (Fathy et al. 2023)	1

### B. Reported challenges

The ERAS protocols presented numerous challenges. There was a lack of standardization of practice (*n* = 2) (Oodit et al. 2021; Ammar et al. 2023), hindering uniform implementation. There were challenges in adhering to the recommended protocols (*n* = 5) (Ammar et al. 2023; Beukes et al. 2022; Elgamal et al. 2023; Abdelrazik and Sanad 2023; Sameer et al. 2023). Limitations in research design and methodologies compromising the ability to generate strong evidence were acknowledged (*n* = 3) (Elgamal et al. 2023; Abdelrazik and Sanad 2023; Sarhan et al. 2023). A lack of trained staff (*n* = 2) (Beukes et al. 2022; Elsabbagh et al. 2023), patient education (*n* = 1) (Abdelrazik and Sanad 2023), and multidisciplinary collaboration (*n* = 3) (Baluku et al. 2023; Abdelrazik and Sanad 2023; Sameer et al. 2023) were also reported as challenges to optimize ERAS effectiveness. Resistance to change (*n* = 3) (Plenge et al. 2023; Beukes et al. 2022; Baluku et al. 2023) posed a barrier to widespread acceptance, while difficulty conducting preoperative optimization (*n* = 1) (Ahmed et al. 2023), and resource limitations (*n* = 1) (Ahmed et al. 2023), was additional challenges. Table 3 shows the reported challenges.

**Table 3:** Reported challenges

Challenges in adopting the protocol	Number of articles
Adherence to protocols (Loots et al. 2023; Ammar et al. 2023; Mohamed Ibrahim et al. 2023; Ahmed et al. 2023; Abdelrazik and Sanad 2023)	5
Multidisciplinary collaboration (Plenge et al. 2023; Ahmed et al. 2023; Abdelrazik and Sanad 2023)	3
Resistance to change (Oodit et al. 2021; Ammar et al. 2023; Plenge et al. 2023)	3
Research design limitation (Mohamed Ibrahim et al. 2023; Ahmed et al. 2023; Elgamal et al. 2023)	3
Lacked standardization (Fathy et al. 2023; Loots et al. 2023)	2
Lack of trained staff and awareness (Ammar et al. 2023; Plenge et al. 2023)	2
Patient education (Ahmed et al. 2023)	1
Difficulty of conducting preoperative optimization (Elsabbagh et al. 2023)	1
Resource limitation (Elsabbagh et al. 2023)	1

### C. Recommendations

Several recommendations were made in the reviewed publications to aid adoption and implementation of ERAS protocols in Africa. Firstly, there was a call for the practical implementation of ERAS principles within surgical practices (*n* = 5) (Fathy et al. 2023; Ammar et al. 2023; Ferghali et al. 2023; Mohamed Ibrahim et al. 2023; Ahmed et al. 2023). Additionally, it was recommended that ERAS should not merely remain a set of principles but should be formally adopted and established as a standard of care in medical practices, with four studies supporting this proposition (*n* = 4) (Plenge et al. 2023; Beukes et al. 2022; Baluku et al. 2023; Abdelrazik and Sanad 2023). Furthermore, studies advocated for a sustained commitment to research efforts, encompassing the evaluation of ERAS effectiveness, potential modifications for improved outcomes, and exploration of its applicability in different surgical contexts (*n* = 8) (Loots et al. 2023; Oodit et al. 2021; Ammar et al. 2023; Baluku et al. 2023; Mohamed Ibrahim et al. 2023; Ahmed et al. 2023; Abdelrazik and Sanad 2023; Sarhan et al. 2023). The feasibility and effectiveness of ERAS in low- and middle-income countries (LMICs) were emphasized (*n* = 1) (Loots et al. 2023). There was also a recommendation to extend ERAS implementation to various surgical disciplines across the African continent (*n* = 1) (Loots et al. 2023). The recognition of perioperative care as a distinct sub-specialty was recommended (*n* = 1) (Plenge et al. 2023). There was a call for the undertaking of cost-effectiveness analyses related to ERAS implementation (*n* = 2) (Baluku et al. 2023; Mohamed Ibrahim et al. 2023), aiming to assess the economic implications and benefits associated with adopting these protocols in low-resource environments. Table 4 shows the recommendations made by the authors for future implementation and expansion of ERAS programs in African healthcare settings.

**Table 4:** Reported recommendations

Recommendations	Number of articles
Further research (Hsieh and Shannon 2005; Fathy et al. 2023; Loots et al. 2023; Plenge et al. 2023; Elsabbagh et al. 2023; Ferghali et al. 2023; Ahmed et al. 2023; Elgamal et al. 2023)	8
To implement the protocol (Peters et al. 2023b; Loots et al. 2023; Baluku et al. 2023; Elsabbagh et al. 2023; Ferghali et al. 2023)	5
Protocol be adopted as a standard of care (Oodit et al. 2021; Ammar et al. 2023; Plenge et al. 2023; Ahmed et al. 2023)	4
Education and training (Ammar et al. 2023; Abdelrazik and Sanad 2023)	2
Continuous evaluation (Ammar et al. 2023; Abdelrazik and Sanad 2023)	2
To conduct a cost-effectiveness analysis (Plenge et al. 2023; Elsabbagh et al. 2023)	2
Team collaboration (Abdelrazik and Sanad 2023)	1
ERAS is feasible and effective in a LMICs (Hsieh and Shannon 2005)	1
ERAS could be implemented for other forms of surgery in South Africa and across the African context (Hsieh and Shannon 2005)	1
Recognition of perioperative care as a sub-specialty (Oodit et al. 2021)	1
Specialty-specific tailored protocol (Plenge et al. 2023)	1

## Discussion

The principal findings of this review are that ERAS protocols can improve patient recovery and postoperative outcomes in African healthcare settings. Although the number of studies is limited, the studies included in this review showed positive impacts of ERAS implementation, including reduced hospital length of stay, lower mortality rates, and improved pain management of patients. However, challenges such as lack of adherence to protocols, standardization, and trained staff were identified, and further research was recommended to understand the potential benefits and barriers to implementing ERAS protocols in African healthcare settings fully.

Despite using an inclusive and comprehensive search strategy, the number of existing studies on the implementation of ERAS protocols in Africa is very low, as we were only able to find a few studies, and they were limited to certain geographical locations, as most of these studies were conducted in Egypt and South Africa. This finding is also consistent with a study on the global distribution of ERAS research, which found that most studies have been conducted in high-income countries (Su et al. 2000). Furthermore, the implementation efforts were mostly in obstetric and gynecological procedures. While this is a positive step, as most surgical procedures are obstetric in Africa (Bentounsi et al. 2023; Biccard et al. 2023a), it is important to increase these studies towards other Bellwether surgeries, such as emergency laparotomy and treatment of open fractures (O’Neill et al. 2023). The capacity to perform these Bellwether procedures is related to performing all obstetric, general, basic, emergency, and orthopedic procedures, and the findings gained from the implementation of ERAS practices might also easily extend to a broader group of surgical procedures (O’Neill et al. 2023).

The findings presented in this review highlight the multifaceted benefits of ERAS protocols in improving various aspects of patient outcomes and healthcare resource utilization. One notable improvement is the consistent reduction in hospital length of stay across the studies, suggesting a more efficient recovery process facilitated by ERAS. This not only contributes to cost savings but also aligns with the broader healthcare priority of minimizing waste and optimizing resource utilization (Zlaugotne et al. 2022), especially in Africa’s healthcare system, where resources are often limited and in high demand (Lavy et al. 2023; Mehta et al. 2023; Frimpong-Boateng and Edwin 2023). The decrease in mortality rates, lower morbidity, and reduced need for readmission highlight the positive impact of implementing ERAS on a large scale in Africa, where high mortality and morbidity rates exist (Biccard et al. 2023a). ERAS protocols can help address these challenges by improving patient outcomes and reducing the burden on healthcare systems.

There were several challenges identified in ERAS protocol implementation that highlight the difficulties of optimizing ERAS practices in African clinical settings. The lack of standardization presents a significant hurdle, impeding consistent application across diverse healthcare contexts. A proposed guideline for ERAS implementation in LMICs could assist in addressing some of these challenges (Oodit et al. 2023). Other challenges related to resistance to change and adherence to ERAS protocol are inevitable challenges of change management (MacPhee 2007), also reported globally (Kehlet 2023). However, overcoming resistance to change and ensuring adherence to protocols will require multidisciplinary collaboration, leadership, and evidence-based strategies (Tippireddy and Ghatol 2023; Gramlich et al. 2024). Even though these are mentioned as some of the challenges, they are also considered recommendations and hence should be part of the solution. In addition, providing adequate training and education to healthcare providers and providing continuous patient education can help in effectively implementing ERAS protocols and ensuring their long-term success. The limitations in research design emphasize the need for robust studies to inform high-level evidence-based practices for ERAS in Africa.

This review suggests that the practical integration and formal adoption of ERAS principles within surgical practices are crucial to improving patient outcomes, which is also supported by another review conducted to assess the feasibility of LMICs, which suggests the utilization of implementation sciences (Riad et al. 2023). This formal adoption would ensure consistent implementation of ERAS principles across different surgical practices, leading to improved patient outcomes and reduced variability in care. This review suggests that this needs to be supported by ongoing education and training of healthcare professionals to effectively implement and sustain ERAS protocols in clinical settings.

There is a general recommendation for the need for further research to gather more evidence to support ERAS in Africa. This was also highlighted as one of the national perioperative research priorities in South Africa, where a stepped-wedge trial of an ERAS program for surgical, obstetric, and trauma procedures was recommended (Biccard et al. 2023b). The authors of this article also stress the importance of conducting well-designed studies with larger sample sizes to provide more robust evidence on the effectiveness and safety of ERAS protocols in diverse clinical settings and surgical procedures. They also recommend that future research focus on evaluating the cost-effectiveness and long-term outcomes associated with implementing these protocols, either fully or partially.

The limitation of this review is as it is a scoping review, and it did not include a comprehensive analysis of all available literature on ERAS programs. Additionally, the studies primarily focused on short-term outcomes and did not thoroughly examine potential complications or adverse events associated with ERAS protocols. Further research is needed to address these gaps in knowledge and provide a more comprehensive understanding of the benefits and limitations of implementing ERAS programs in various healthcare settings.

## Conclusion

In conclusion, ERAS protocols have shown promising results in improving patient outcomes and optimizing healthcare resource utilization in African surgical settings. This indicates the potential to contribute to a patient-centered approach by reducing complications, shortening hospital stays, and enhancing the overall patient experience during the surgical process. While there are limited studies available, along with different challenges, the positive impacts observed justify further exploration and investment in implementing ERAS. It is essential to expand research to cover a wider range of surgical procedures and geographic locations to establish a strong evidence base.

### Supplementary Information

Supplementary Material 1: Appendix 1. Search strategies.

Supplementary Material 2: Appendix 2. Data extraction tool.

## Data Availability

No datasets were generated or analysed during the current study.
