# Supplementary material for: A scoping review of Enhanced Recovery After Surgery (ERAS), protocol implementation, and its impact on surgical outcomes and healthcare systems in Africa
Source: Perioper Med (Lond). 2024 Aug 2;13:86. doi: 10.1186/s13741-024-00435-2 (PMC11297632; doi:10.1186/s13741-024-00435-2)
Supplement: Supplementary file 1 — Supplementary Material 1: Appendix 1. Search strategies. [file 13741_2024_435_MOESM1_ESM.docx]

Appendix 1: Search strategies

Enhanced Recovery After Surgery (ERAS) in Africa,

PubMed

|  | MESH | Keywords | TextWord |
| --- | --- | --- | --- |
|  | "Enhanced Recovery After Surgery"[Mesh] | Enhanced Recovery After Surgery OR ERAS OR fast-track surgery OR fast track surgery FAST OR Enhanced Postsurgical Recovery |  |
|  |  |  |  |
|  |  |  |  |
| Filter: Africa |  |  |  |

Searched PubMed on October 2023

| Search | Query | Records retrieved |
| --- | --- | --- |
| #1 | **"Enhanced Recovery After Surgery"[Mesh]** | 1490 |
| #2 | Enhanced Recovery After Surgery OR ERAS OR fast-track surgery OR fast track surgery FAST OR Enhanced Postsurgical Recovery | 14,771 |
| #3 |  |  |
| Filter |  |  |
|  |  | 213 |

| <https://www.ncbi.nlm.nih.gov/sites/myncbi/1LwlVrv_cv_5m/collections/63278639/public/> |
| --- |

| #9 |  |
| --- | --- |

| Search: (("Enhanced Recovery After Surgery"[Mesh]) OR (Enhanced Recovery After Surgery OR ERAS OR fast-track surgery OR fast track surgery FAST OR Enhanced Postsurgical Recovery)) AND ((Africa [MeSH]) OR (Africa[Text Word] OR African[Text Word] OR Algeria[Text Word] OR Angola[Text Word] OR Benin[Text Word] OR Botswana[Text Word] OR "Burkina Faso"[Text Word] OR Burundi[Text Word] OR "Cabo Verde"[Text Word] OR Cameroon[Text Word] OR Cameroun[Text Word] OR "Canary Islands"[Text Word] OR "Cape Verde"[Text Word] OR "Central African Republic"[Text Word] OR Chad[Text Word] OR Comoros[Text Word] OR Congo[Text Word] OR "Cote d'Ivoire"[Text Word] OR "Democratic Republic of Congo"[Text Word] OR Djibouti[Text Word] OR Egypt[Text Word] OR Eritrea[Text Word] OR eSwatini[Text Word] OR Ethiopia[Text Word] OR Gabon[Text Word] OR Gambia[Text Word] OR Ghana[Text Word] OR Guinea[Text Word] OR Guinea- Bissau[Text Word] OR "Ivory Coast"[Text Word] OR Jamahiriya[Text Word] OR Kenya[Text Word] OR Lesotho[Text Word] OR Liberia[Text Word] OR Libya[Text Word] OR Madagascar[Text Word] OR Malawi[Text Word] OR Mali[Text Word] OR Mauritania[Text Word] OR Mauritius[Text Word] OR Mayotte[Text Word] OR Morocco[Text Word] OR Mozambique[Text Word] OR Namibia[Text Word] OR Niger[Text Word] OR Nigeria[Text Word] OR Principe[Text Word] OR Reunion[Text Word] OR Rwanda[Text Word] OR "Saint Helena"[Text Word] OR "Sao Tome"[Text Word] OR Senegal[Text Word] OR Seychelles[Text Word] OR "Sierra Leone"[Text Word] OR Somalia[Text Word] OR "St Helena"[Text Word] OR Sudan[Text Word] OR Swaziland[Text Word] OR Tanzania[Text Word] OR Togo[Text Word] OR Tunisia[Text Word] OR Uganda[Text Word] OR "Western Sahara"[Text Word] OR Zaire[Text Word] OR Zambia[Text Word] OR Zimbabwe[Text Word])) | [213](https://pubmed.ncbi.nlm.nih.gov/?term=longquery94754e8863fbcae02c4c&sort=relevance&size=200) | 08:47:43 |
| --- | --- | --- |
| #8 |  |  |

| Search: (Africa [MeSH]) OR (Africa[Text Word] OR African[Text Word] OR Algeria[Text Word] OR Angola[Text Word] OR Benin[Text Word] OR Botswana[Text Word] OR "Burkina Faso"[Text Word] OR Burundi[Text Word] OR "Cabo Verde"[Text Word] OR Cameroon[Text Word] OR Cameroun[Text Word] OR "Canary Islands"[Text Word] OR "Cape Verde"[Text Word] OR "Central African Republic"[Text Word] OR Chad[Text Word] OR Comoros[Text Word] OR Congo[Text Word] OR "Cote d'Ivoire"[Text Word] OR "Democratic Republic of Congo"[Text Word] OR Djibouti[Text Word] OR Egypt[Text Word] OR Eritrea[Text Word] OR eSwatini[Text Word] OR Ethiopia[Text Word] OR Gabon[Text Word] OR Gambia[Text Word] OR Ghana[Text Word] OR Guinea[Text Word] OR Guinea- Bissau[Text Word] OR "Ivory Coast"[Text Word] OR Jamahiriya[Text Word] OR Kenya[Text Word] OR Lesotho[Text Word] OR Liberia[Text Word] OR Libya[Text Word] OR Madagascar[Text Word] OR Malawi[Text Word] OR Mali[Text Word] OR Mauritania[Text Word] OR Mauritius[Text Word] OR Mayotte[Text Word] OR Morocco[Text Word] OR Mozambique[Text Word] OR Namibia[Text Word] OR Niger[Text Word] OR Nigeria[Text Word] OR Principe[Text Word] OR Reunion[Text Word] OR Rwanda[Text Word] OR "Saint Helena"[Text Word] OR "Sao Tome"[Text Word] OR Senegal[Text Word] OR Seychelles[Text Word] OR "Sierra Leone"[Text Word] OR Somalia[Text Word] OR "St Helena"[Text Word] OR Sudan[Text Word] OR Swaziland[Text Word] OR Tanzania[Text Word] OR Togo[Text Word] OR Tunisia[Text Word] OR Uganda[Text Word] OR "Western Sahara"[Text Word] OR Zaire[Text Word] OR Zambia[Text Word] OR Zimbabwe[Text Word]) | [781,398](https://pubmed.ncbi.nlm.nih.gov/?term=longquery9ba587ee9eaaa5380ae7&sort=relevance&size=200) | 08:47:20 |
| --- | --- | --- |
| #7 |  |  |

| Search: Africa[Text Word] OR African[Text Word] OR Algeria[Text Word] OR Angola[Text Word] OR Benin[Text Word] OR Botswana[Text Word] OR "Burkina Faso"[Text Word] OR Burundi[Text Word] OR "Cabo Verde"[Text Word] OR Cameroon[Text Word] OR Cameroun[Text Word] OR "Canary Islands"[Text Word] OR "Cape Verde"[Text Word] OR "Central African Republic"[Text Word] OR Chad[Text Word] OR Comoros[Text Word] OR Congo[Text Word] OR "Cote d'Ivoire"[Text Word] OR "Democratic Republic of Congo"[Text Word] OR Djibouti[Text Word] OR Egypt[Text Word] OR Eritrea[Text Word] OR eSwatini[Text Word] OR Ethiopia[Text Word] OR Gabon[Text Word] OR Gambia[Text Word] OR Ghana[Text Word] OR Guinea[Text Word] OR Guinea- Bissau[Text Word] OR "Ivory Coast"[Text Word] OR Jamahiriya[Text Word] OR Kenya[Text Word] OR Lesotho[Text Word] OR Liberia[Text Word] OR Libya[Text Word] OR Madagascar[Text Word] OR Malawi[Text Word] OR Mali[Text Word] OR Mauritania[Text Word] OR Mauritius[Text Word] OR Mayotte[Text Word] OR Morocco[Text Word] OR Mozambique[Text Word] OR Namibia[Text Word] OR Niger[Text Word] OR Nigeria[Text Word] OR Principe[Text Word] OR Reunion[Text Word] OR Rwanda[Text Word] OR "Saint Helena"[Text Word] OR "Sao Tome"[Text Word] OR Senegal[Text Word] OR Seychelles[Text Word] OR "Sierra Leone"[Text Word] OR Somalia[Text Word] OR "St Helena"[Text Word] OR Sudan[Text Word] OR Swaziland[Text Word] OR Tanzania[Text Word] OR Togo[Text Word] OR Tunisia[Text Word] OR Uganda[Text Word] OR "Western Sahara"[Text Word] OR Zaire[Text Word] OR Zambia[Text Word] OR Zimbabwe[Text Word] | [781,398](https://pubmed.ncbi.nlm.nih.gov/?term=longquery1c5b7f20a225a80134b6&sort=relevance&size=200) | 08:46:50 |
| --- | --- | --- |
| #6 |  |  |

| Search: Africa [MeSH] | [327,513](https://pubmed.ncbi.nlm.nih.gov/?term=Africa+%5BMeSH%5D&sort=relevance&size=200) | 08:46:27 |
| --- | --- | --- |
| #5 |  |  |

| Search: ("Enhanced Recovery After Surgery"[Mesh]) OR (Enhanced Recovery After Surgery OR ERAS OR fast-track surgery OR fast track surgery FAST OR Enhanced Postsurgical Recovery) | [14,771](https://pubmed.ncbi.nlm.nih.gov/?term=%28%22Enhanced+Recovery+After+Surgery%22%5BMesh%5D%29+OR+%28Enhanced+Recovery+After+Surgery+OR+ERAS+OR+fast-track+surgery+OR+fast+track+surgery+FAST+OR+Enhanced+Postsurgical+Recovery%29&sort=relevance&size=200) | 08:45:35 |
| --- | --- | --- |
| #4 |  |  |

| Search: Enhanced Recovery After Surgery OR ERAS OR fast-track surgery OR fast track surgery FAST OR Enhanced Postsurgical Recovery | [14,771](https://pubmed.ncbi.nlm.nih.gov/?term=Enhanced+Recovery+After+Surgery+OR+ERAS+OR+fast-track+surgery+OR+fast+track+surgery+FAST+OR+Enhanced+Postsurgical+Recovery&sort=relevance&size=200) | 08:45:17 |
| --- | --- | --- |
| #3 |  |  |

| Search: "Enhanced Recovery After Surgery"[Mesh] Sort by: Most Recent | [1,490](https://pubmed.ncbi.nlm.nih.gov/?sort=date&term=%22Enhanced+Recovery+After+Surgery%22%5BMesh%5D&size=200) | 08:43:07 |
| --- | --- | --- |

Scopus

| TITLE-ABS-KEY ( "Enhanced Recovery After Surgery" OR "fast-track surgery" OR "fast track surgery" OR "Enhanced Postsurgical Recovery" )  [7,049 results](https://www.scopus.com/search/history/results.uri?origin=searchhistory&shid=8) |  |
| --- | --- |
| TITLE-ABS-KEY ( africa OR african OR algeria OR angola OR benin OR botswana OR "Burkina Faso" OR burundi OR "Cabo Verde" OR cameroon OR cameroun OR "Canary Islands" OR "Cape Verde" OR "Central African Republic" OR chad OR comoros OR congo OR "Cote d'Ivoire" OR "Democratic Republic of Congo" OR djibouti OR egypt OR eritrea OR eswatini OR ethiopia OR gabon OR gambia OR ghana OR guinea OR guinea- AND bissau OR "Ivory Coast" OR jamahiriya OR kenya OR lesotho OR liberia OR libya OR madagascar OR malawi OR mali OR mauritania OR mauritius OR mayotte OR morocco OR mozambique OR namibia OR niger OR nigeria OR principe OR reunion OR rwanda OR "Saint Helena" OR "Sao Tome" OR senegal OR seychelles OR "Sierra Leone" OR somalia OR "St Helena" OR sudan OR swaziland OR tanzania OR togo OR tunisia OR uganda OR "Western Sahara" OR zaire OR zambia OR zimbabwe ) |  |

| “Enhanced Recovery After Surgery” OR ERAS OR “fast-track surgery” OR “fast track surgery” FAST OR “Enhanced Postsurgical Recovery” |  |
| --- | --- |
| Africa OR African OR Algeria OR Angola OR Benin OR Botswana OR "Burkina Faso" OR Burundi OR “Cabo Verde” OR Cameroon OR Cameroun OR "Canary Islands" OR "Cape Verde" OR "Central African Republic" OR Chad OR Comoros OR Congo OR "Cote d'Ivoire" OR "Democratic Republic of Congo" OR Djibouti OR Egypt OR Eritrea OR eSwatini OR Ethiopia OR Gabon OR Gambia OR Ghana OR Guinea OR Guinea- Bissau OR "Ivory Coast" OR Jamahiriya OR Kenya OR Lesotho OR Liberia OR Libya OR Madagascar OR Malawi OR Mali OR Mauritania OR Mauritius OR Mayotte OR Morocco OR Mozambique OR Namibia OR Niger OR Nigeria OR Principe OR Reunion OR Rwanda OR “Saint Helena” OR “Sao Tome” OR Senegal OR Seychelles OR “Sierra Leone” OR Somalia OR “St Helena” OR Sudan OR Swaziland OR Tanzania OR Togo OR Tunisia OR Uganda OR “Western Sahara” OR Zaire OR Zambia OR Zimbabwe |  |

( ALL ( "Enhanced Recovery After Surgery" OR "fast-track surgery" OR "fast track surgery" OR "Enhanced Postsurgical Recovery" ) ) AND ( ALL ( africa OR african OR algeria OR angola OR benin OR botswana OR "Burkina Faso" OR burundi OR "Cabo Verde" OR cameroon OR cameroun OR "Canary Islands" OR "Cape Verde" OR "Central African Republic" OR chad OR comoros OR congo OR "Cote d'Ivoire" OR "Democratic Republic of Congo" OR djibouti OR egypt OR eritrea OR eswatini OR ethiopia OR gabon OR gambia OR ghana OR guinea OR guinea- AND bissau OR "Ivory Coast" OR jamahiriya OR kenya OR lesotho OR liberia OR libya OR madagascar OR malawi OR mali OR mauritania OR mauritius OR mayotte OR morocco OR mozambique OR namibia OR niger OR nigeria OR principe OR reunion OR rwanda OR "Saint Helena" OR "Sao Tome" OR senegal OR seychelles OR "Sierra Leone" OR somalia OR "St Helena" OR sudan OR swaziland OR tanzania OR togo OR tunisia OR uganda OR "Western Sahara" OR zaire OR zambia OR zimbabwe ) )

[151 results](https://www.scopus.com/search/history/results.uri?origin=searchhistory&shid=15)



ALL ( "Enhanced Recovery After Surgery" OR "fast-track surgery" OR "fast track surgery" OR "Enhanced Postsurgical Recovery" )

[22,498 results](https://www.scopus.com/search/history/results.uri?origin=searchhistory&shid=14)



 13

[Edit](https://www.scopus.com/search/history/edit.uri?shid=13)

( TITLE-ABS-KEY ( "Enhanced Recovery After Surgery" OR "fast-track surgery" OR "fast track surgery" OR "Enhanced Postsurgical Recovery" ) ) AND ( ALL ( africa OR african OR algeria OR angola OR benin OR botswana OR "Burkina Faso" OR burundi OR "Cabo Verde" OR cameroon OR cameroun OR "Canary Islands" OR "Cape Verde" OR "Central African Republic" OR chad OR comoros OR congo OR "Cote d'Ivoire" OR "Democratic Republic of Congo" OR djibouti OR egypt OR eritrea OR eswatini OR ethiopia OR gabon OR gambia OR ghana OR guinea OR guinea- AND bissau OR "Ivory Coast" OR jamahiriya OR kenya OR lesotho OR liberia OR libya OR madagascar OR malawi OR mali OR mauritania OR mauritius OR mayotte OR morocco OR mozambique OR namibia OR niger OR nigeria OR principe OR reunion OR rwanda OR "Saint Helena" OR "Sao Tome" OR senegal OR seychelles OR "Sierra Leone" OR somalia OR "St Helena" OR sudan OR swaziland OR tanzania OR togo OR tunisia OR uganda OR "Western Sahara" OR zaire OR zambia OR zimbabwe ) )

[27 results](https://www.scopus.com/search/history/results.uri?origin=searchhistory&shid=13)



 11

[Edit](https://www.scopus.com/search/history/edit.uri?shid=11)

ALL ( africa OR african OR algeria OR angola OR benin OR botswana OR "Burkina Faso" OR burundi OR "Cabo Verde" OR cameroon OR cameroun OR "Canary Islands" OR "Cape Verde" OR "Central African Republic" OR chad OR comoros OR congo OR "Cote d'Ivoire" OR "Democratic Republic of Congo" OR djibouti OR egypt OR eritrea OR eswatini OR ethiopia OR gabon OR gambia OR ghana OR guinea OR guinea- AND bissau OR "Ivory Coast" OR jamahiriya OR kenya OR lesotho OR liberia OR libya OR madagascar OR malawi OR mali OR mauritania OR mauritius OR mayotte OR morocco OR mozambique OR namibia OR niger OR nigeria OR principe OR reunion OR rwanda OR "Saint Helena" OR "Sao Tome" OR senegal OR seychelles OR "Sierra Leone" OR somalia OR "St Helena" OR sudan OR swaziland OR tanzania OR togo OR tunisia OR uganda OR "Western Sahara" OR zaire OR zambia OR zimbabwe )

[1,517,191 results](https://www.scopus.com/search/history/results.uri?origin=searchhistory&shid=11)



 8

[Edit](https://www.scopus.com/search/history/edit.uri?shid=8)

TITLE-ABS-KEY ( "Enhanced Recovery After Surgery" OR "fast-track surgery" OR "fast track surgery" OR "Enhanced Postsurgical Recovery" )

[7,049 results](https://www.scopus.com/search/history/results.uri?origin=searchhistory&shid=8)



 2

[Edit](https://www.scopus.com/search/history/edit.uri?shid=2)

TITLE-ABS-KEY ( africa OR african OR algeria OR angola OR benin OR botswana OR "Burkina Faso" OR burundi OR "Cabo Verde" OR cameroon OR cameroun OR "Canary Islands" OR "Cape Verde" OR "Central African Republic" OR chad OR comoros OR congo OR "Cote d'Ivoire" OR "Democratic Republic of Congo" OR djibouti OR egypt OR eritrea OR eswatini OR ethiopia OR gabon OR gambia OR ghana OR guinea OR guinea- AND bissau OR "Ivory Coast" OR jamahiriya OR kenya OR lesotho OR liberia OR libya OR madagascar OR malawi OR mali OR mauritania OR mauritius OR mayotte OR morocco OR mozambique OR namibia OR niger OR nigeria OR principe OR reunion OR rwanda OR "Saint Helena" OR "Sao Tome" OR senegal OR seychelles OR "Sierra Leone" OR somalia OR "St Helena" OR sudan OR swaziland OR tanzania OR togo OR tunisia OR uganda OR "Western Sahara" OR zaire OR zambia OR zimbabwe )

[176,103 results](https://www.scopus.com/search/history/results.uri?origin=searchhistory&shid=2)
