# Supplementary material for: A scoping review of Enhanced Recovery After Surgery (ERAS), protocol implementation, and its impact on surgical outcomes and healthcare systems in Africa
Source: Perioper Med (Lond). 2024 Aug 2;13:86. doi: 10.1186/s13741-024-00435-2 (PMC11297632; doi:10.1186/s13741-024-00435-2)
Supplement: Supplementary file 2 — Supplementary Material 2: Appendix 2. Data extraction tool. [file 13741_2024_435_MOESM2_ESM.docx]

| Study ID  *(e.g. author name, year)* | |  | | | | |
| --- | --- | --- | --- | --- | --- | --- |
| Form completed by | |  | | | | |
| Study author contact details | |  | | | | |
| Author and year of publication: | |  | | | | |
| Country | |  | | | | |
| Start & end dates | |  | | | | |
| Possible conflicts of interest | |  | | | | |
| Methods | Type |  | | | | |
|  | **Design** |  | | | | |
|  | **Aim of study** |  | | | | |
|  | **Follow up** |  | | | | |
| Notes: |  | | | | | |
| Participants | **Total no. participant** | |  | | | |
|  |  | | *ERAS* | | | *Routine* |
|  | **Surgical specialty type** | |  | | |  |
| Note |  | | | |  |  |
| Study ID |  | | | |  |  |
|  | Description as stated in report/paper and Location in text or source (pg & ¶/fig/table/other) | | | | | |
| Reported clinical outcomes |  | | | | | |
| Reported Challenges |  | | | | | |
| Key conclusions of study authors |  | | | | | |
| Key Recommendation of study authors |  | | | | | |
| References to other relevant studies |  | | | | | |
| Correspondence required for further study information (from whom, what and when) | | | |  | | |
| Notes: | | | | | | |

Appendix 2: Data extraction tool
